# A Rare Case Report of Non-toxigenic Corynebacterium diphtheriae Bloodstream Infection in an Uncontrolled Diabetic With Peripheral Vascular Disease

**DOI:** 10.7759/cureus.14947

**Published:** 2021-05-10

**Authors:** Lakshmi Shanmugam, Ketan Priyadarshi, Mahalakshmi Kumaresan, Monika Sivaradjy, Praveen Upadhyay, TP Elamurugan, Apurba S Sastry

**Affiliations:** 1 Microbiology, Jawaharlal Institute of Postgraduate Medical Education and Research, Puducherry, IND; 2 Surgery, Jawaharlal Institute of Postgraduate Medical Education and Research, Puducherry, IND; 3 Microbiology: Hospital Infection Control, Jawaharlal Institute of Postgraduate Medical Education and Research, Puducherry, IND

**Keywords:** corynebacterium diphtheriae, non-toxigenic, peripheral vascular disease, uncontrolled diabetes, gravis biotype, vitek 2, maldi tof ms, antimicrobial susceptibility

## Abstract

*Corynebacterium diphtheriae *usually causes respiratory diphtheria, which is considered as a disease of toxemia but never bacteremia. Over the last few decades, cutaneous diphtheria has been increasingly reported owing to the emergence of the non-toxigenic strain, which causes locally necrotic and ulcerative lesions. Bacteremia is very rare, but the existing evidence in the literature suggests that the organism can rarely cause invasive infections such as septicemia, endocarditis, and osteoarthritis. Here, we present a rare case of *C. diphtheriae* causing bloodstream infections in an elderly diabetic with peripheral vascular disease, which was diagnosed incidentally on routine blood culture owing to automated identification systems viz matrix-assisted laser desorption ionization-time of flight (MALDI-TOF) confirmed with conventional methods, and susceptibility was performed using automated VITEK 2 system (BioMérieux, Marcy-l'Étoile, France), which has aided in the timely management.

## Introduction

*Corynebacterium diphtheriae* is an aerobic, gram-positive, non-motile, non-spore-forming, non-capsulated, non-fastidious, club-shaped gram-positive bacillus. The most common clinical presentation of this organism is respiratory diphtheria, a toxin-mediated disease characterized by the formation of pseudomembrane in various respiratory sites. However, over the last few decades, cutaneous diphtheria has been increasingly reported owing to the emergence of the non-toxigenic strain, subsequent to widespread vaccination programs [1].

Traditionally diphtheria is considered a disease of toxemia but never bacteremia. Bacilli are noninvasive, are present only at the localized site (pharynx), and secrete the toxin that spreads via bloodstream to various organs. Cutaneous diphtheria is also locally necrotic and ulcerative. But the existing evidence in the literature suggests that the organism can rarely cause invasive infections such as septicemia, endocarditis, and osteoarthritis [1]. Here, we present a rare case of *C. diphtheriae* causing bloodstream infections in a diabetic individual with peripheral vascular disease.

## Case presentation

**Figure 1:**
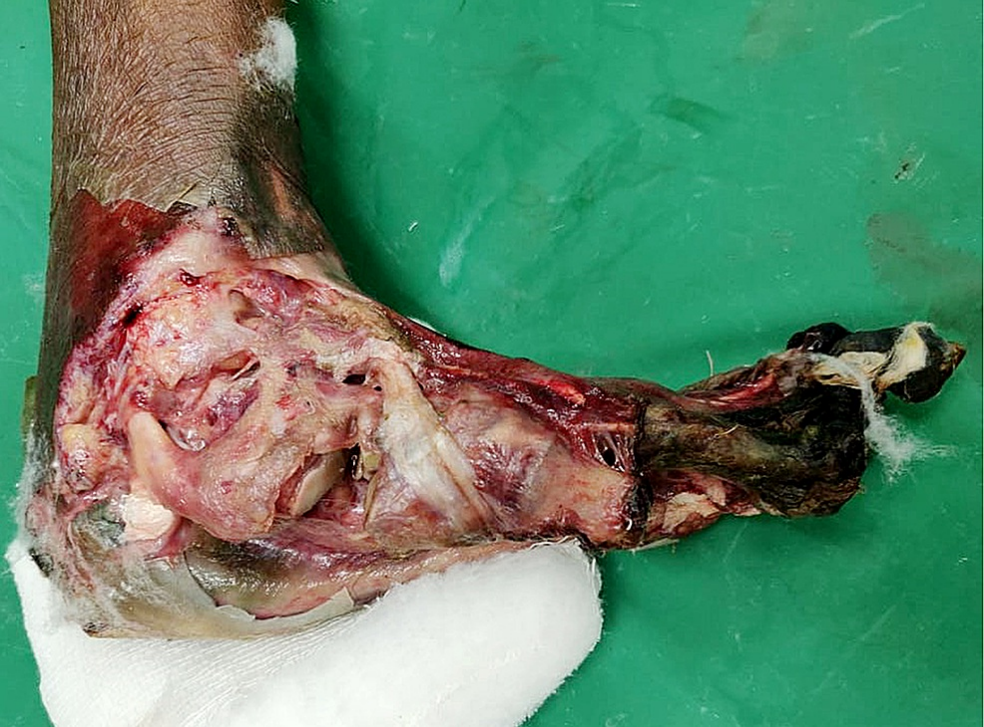
Post debridement status of gangrenous necrotic ulcer over the distal region of left foot

A 63-year-old non-hypertensive diabetic male, farmer by profession, presented with complaints of blackish discoloration of distal part of the left foot for 15 days predominantly involving four small toes. He had similar blackish discoloration of left great toe for last one month, which was amputated 15 days back in a local hospital. He also complained of ulcerative wound over the distal part of left foot and amputated great toe (Figure 1). He was diagnosed as a case of uncontrolled type-2 diabetes mellitus with poor glycemic control on irregular oral hypoglycemics for five years. He had a history of claudication pain in lower limbs for the last one year, which gradually progresses to pain even at rest and loss of sensation of digits for the last three months. He is a chronic smoker and alcoholic for the past 20 years. There was no history of fever, pus discharge from the distal lower limb, chest pain, blurring of vision, blackouts, or abdominal pain.

On clinical examination, there were no signs of pallor, icterus, pedal edema, lymphadenopathy, clubbing, or cyanosis with random blood glucose of 344 mg/dl. Extensive ulceration and necrosis were present over the ankle, heel, and great toe region of left lower limb along with blackish discoloration of second to fifth toes. The peripheral arterial pulses were not palpable in all major peripheral arteries of both lower limbs. Computed tomograghy (CT) angiogram of lower limbs showed high-grade occlusion and luminal narrowing in almost all major arteries of both lower limbs. The provisional diagnosis was made as bilateral lower limb peripheral vascular disease due to uncontrolled diabetes mellitus. The patient was planned for above-knee amputation of left lower limb.

**Figure 2:**
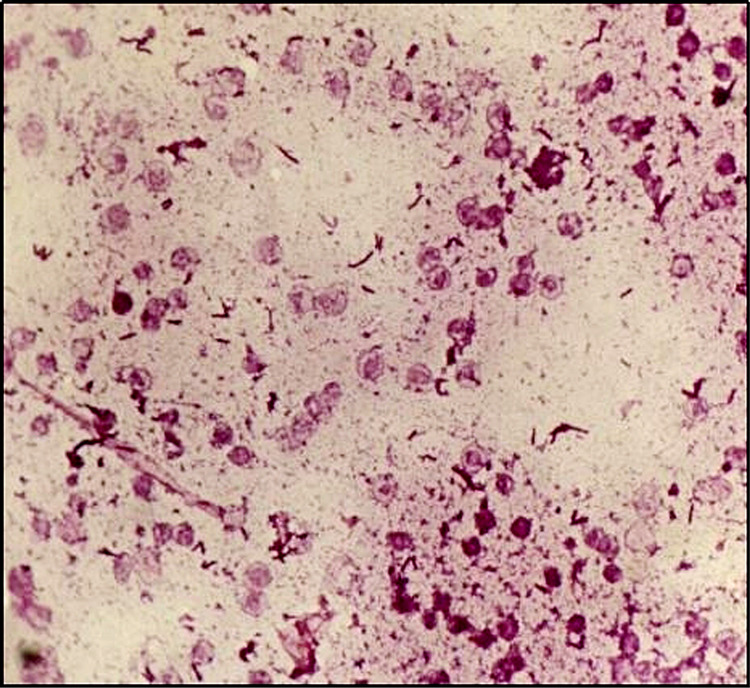
Gram stain from blood culture broth showing club-shaped gram-positive bacilli in cuneiform arrangement

The patient had developed few episodes of mild grade fever, for which automated blood culture was collected and sent to the microbiology laboratory. The bottle was loaded in BacT/Alert Virtuo system (BioMérieux, Marcy-l'Étoile, France), which flagged positive signal after 26 hours of aerobic incubation at 37^o^C. Gram staining was performed directly from flagged broth, which showed short gram-positive bacilli arranged in pairs with acute angular fashion (cuneiform arrangement) as shown in Figure 2.

**Figure 3:**
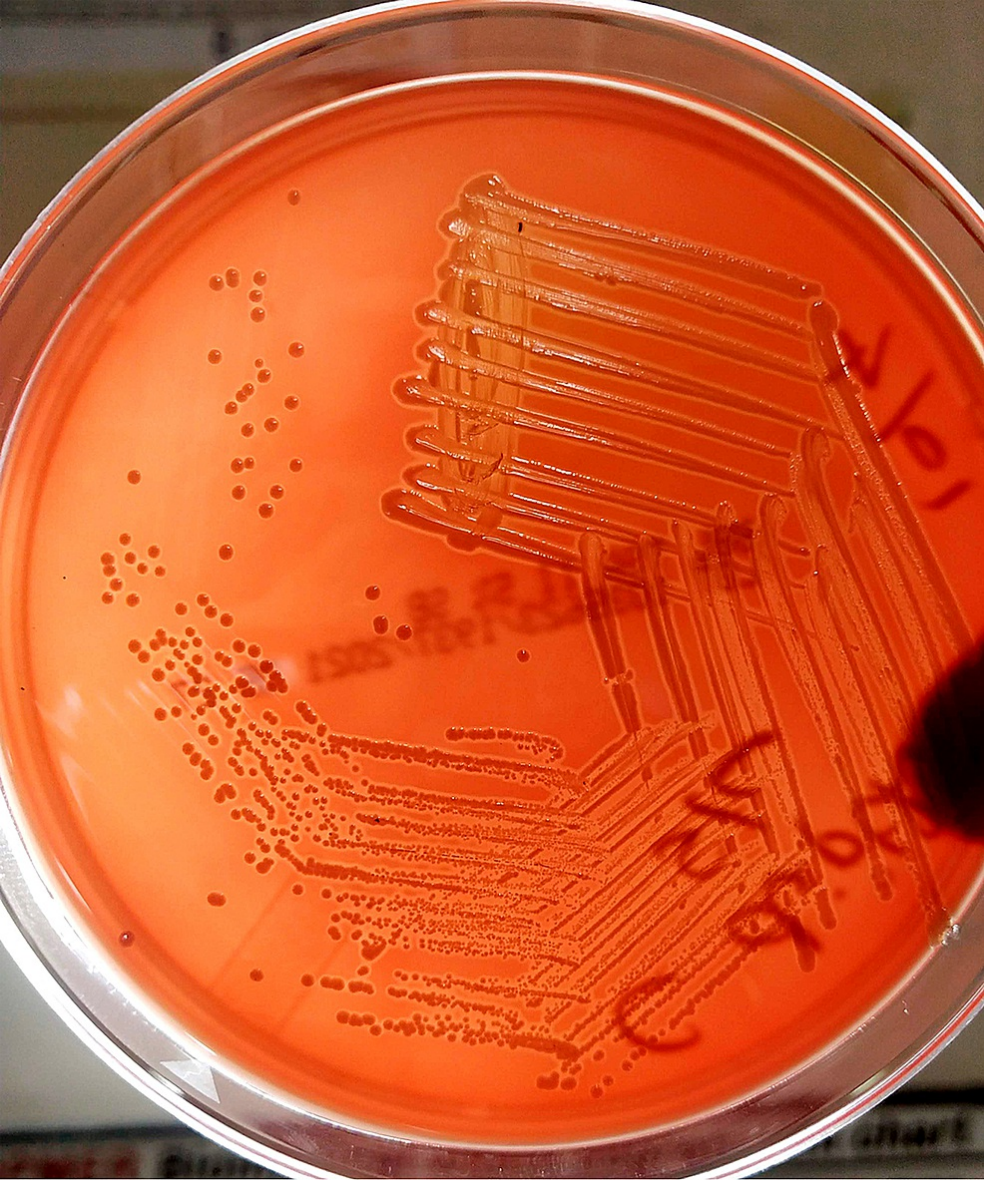
Growth on 5% sheep blood agar showing minute grayish circular low-convex hemolytic colonies

Subculture from bottle was performed on 5% sheep blood agar (SBA), chocolate agar (CA), and MacConkey agar (MAC) and incubated aerobically at 37^o^C. After 10 to 12 hours of incubation, minute grayish circular low-convex hemolytic colonies were seen on SBA (Figure 3). Gram staining from the colonies grown on SBA showed short gram-positive bacilli in cuneiform arrangement with swelling on one or both ends (club-shaped). This is followed by Albert staining from the colony, which showed green-colored bacilli with bluish-purple metachromatic granules in cuneiform arrangement. Identification of the organism from the colony was obtained using matrix-assisted laser desorption ionization-time of flight mass spectrometry (MALDI-TOF MS) (VITEK MS version 3.0). It was identified as *C. diphtheriae* with a confidence interval of 99%. Later, the identification was also confirmed by conventional method.

**Figure 4:**
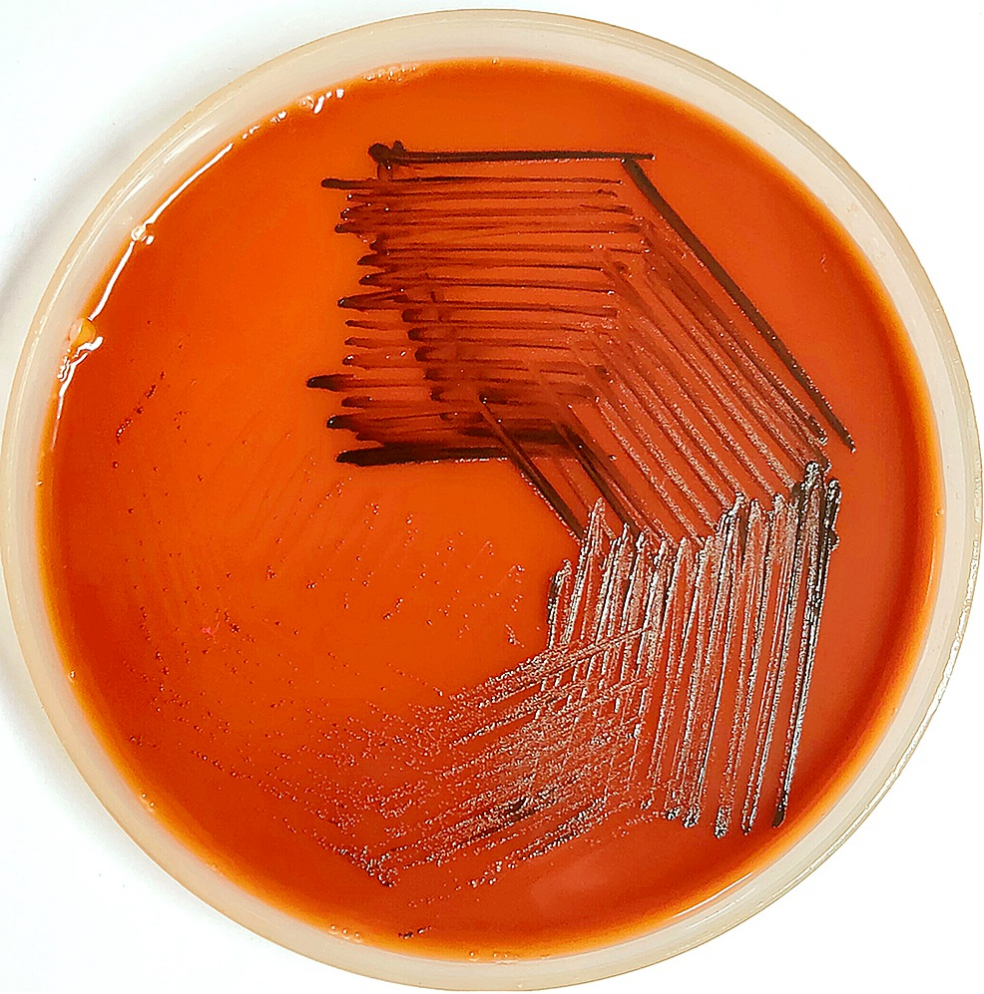
Growth on potassium tellurite blood agar showing jet black colonies

Urea was not hydrolyzed; it produced black-colored colonies on potassium tellurite blood agar (KTBA) and fermented glucose, maltose, and starch with production of acid but no gas on Hiss’s serum sugar fermentation media (Figure 4). The isolate was identified as “gravis” biotype. Antimicrobial susceptibility testing (AST) was performed by broth microdilution method using VITEK-2-automated AST system (AST-P628 card for gram-positive cocci). The result was interpreted using clinical breakpoints given in Clinical and Laboratory Standards Institute (CLSI) M-45 document [2]. The isolate was susceptible for benzylpenicillin, erythromycin, gentamicin, vancomycin, clindamycin, linezolid, and rifampicin, intermediate to ciprofloxacin and resistant to cotrimoxazole and tetracycline. In-house conventional PCR for *tox* gene (*tox A* and *tox B*) and *dtxR* gene was performed from the culture growth. The isolate was negative for* tox *genes but positive for *dtxR* gene, which confirms that the isolate belongs to a non-toxigenic strain of *C. diphtheriae*, biotype gravis.

Following amputation, intra-operative necrotic tissue bits were sent for bacterial culture, which grew* Providencia rettgeri* and *Enterococcus faecalis*. The laboratory failed to show any growth of *C. diphtheriae* from tissue bit, possibly due to heavy gram-negative bacilli growth. Subsequently, a culture of throat swab specimen was also performed to find out any colonization, which failed to have any growth of *C. diphtheriae*. Therefore, the source of* C. diphtheriae* bacteremia was not properly established. The patient was empirically started on amikacin as pre-surgical prophylaxis since admission and subsequently changed to amoxycillin-clavulanate and clindamycin based on the AST report. The patient improved clinically and was discharged on the same regimen.

## Discussion

Diphtheria is generally a non-invasive disease characterized by toxemia but never bacteremia. However, the isolation of *C. diphtheriae* in blood, though rare, has always been regarded as significant in the existing literature. 

There have been case reports of *C. diphtheriae* septicemia [1,3,4], infective endocarditis (native valve [5] and prosthetic valve [6]), and septic arthritis [7] due to non-toxigenic strains among varied age-groups viz child [1,4], adults [3], and risk-groups viz smokers [3], acute myeloid leukemia [1], congenital cardiopathy [4], etc. Only 58 cases of bacteremia infections were described between 1893 and 2003 due to this organism [8].

The most important virulence factor of *C. diphtheriae* is diphtheria toxin (DT). However, the invasive infections are mostly caused by non-toxigenic strains, which suggests that there could be other possible virulence factors such as adhesins, haemagglutinins, and surface-exposed non-fimbrial proteins. Extensive diphtheria vaccination programs (78% DPT vaccine coverage rate in India [8]) that aim at reducing toxin-mediated diseases has created a selection pressure resulting in the acquisition of other virulence factors like transposons, plasmids, etc. that might contain isolated virulence genes or pathogenicity island as a whole [3]. There are in-vitro studies, which suggest that invasiveness is because of a zipper-like mechanism mediated by some cell surface receptors, the details of which remain unclear [9].

In past, identification of *C. diphtheriae* was solely based on conventional methods such as morphological appearance, Albert stain findings, growth on KTBA, and biochemical properties are now strengthened by automated identification methods like MALDI-TOF MS [3]. Here, we have used a combination of the conventional method and MALDI-TOF MS for the identification and biotyping of *C. diphtheriae*. Available literature says that the accuracy of the MALDI-TOF MS system for the identification of *C. diphtheriae, C. pseudotuberculosis,* and *C. ulcerans* is very high (97%-100%) [9]. Toxin detection can be done by Elek’s gel precipitation test [1,3] or PCR [3] for detection of genes such as *tox, dtxR, *16S ribosomal RNA (*16S rRNA*), RNA polymerase β subunit (*rpoB*), etc. We have performed conventional PCR for *tox *and gene. We have determined minimum inhibitory concentration (MIC) by VITEK-2-automated AST system and interpreted using CLSI MIC breakpoints, similar to Wojewoda et al. who have determined MIC using microbroth dilution method [1,3].

This is a rare case of non-toxigenic *C. diphtheriae *bloodstream infection (BSI) in a diabetic individual with peripheral vascular disease. In the present case, the patient was having risk factors like diabetes mellitus and peripheral vascular disease with gangrenous lower limb ulcers. The postulated hypotheses are as follows: (i) organisms gain access from the environment into the poorly vascularized gangrenous limb with breached skin barrier, multiply in the site, and cause extensive necrosis of the distal lower limb and subsequent entry into the bloodstream. (ii) BSI can be preceded with asymptomatic colonization of the upper respiratory tract in an apparently immunocompromised host. But in this study, we failed to attribute the source of BSI to either of these, as the culture of both throat swab and tissue bit from the wound did not yield *C. diphtheriae*. The possible reasons may be (i) prior institution of susceptible antimicrobial therapy viz amikacin before sending culture samples or (ii) the wound tissue had a heavy growth of a mixture of gram-negative organisms on SBA and MAC, because of which *C. diphtheriae *could not have been isolated. (iii) Repeat sampling was not possible because the patient underwent amputation.

## Conclusions

This case highlights an unusual presentation of non-toxigenic *C. diphtheriae* bacteremia in a patient with immunocompromised comorbidities. This emphasizes sending appropriate clinical samples for microbiological cultures including blood culture before the institution of empirical antimicrobial therapy. The present case is a laboratory-guided incidental finding of* C. diphtheriae* BSI, most possibly preceded by cutaneous diphtheria. Automated identification and AST system help in arriving at an accurate and timely diagnosis for the appropriate management of the patient.
